# Unleashing the potential of metaphors: a categorization system for exploring return to work after maternity

**DOI:** 10.1007/s00737-024-01446-0

**Published:** 2024-02-20

**Authors:** Sebastiano Rapisarda, Valentina Santoro, Laura Dal Corso

**Affiliations:** Department of Philosophy, Sociology, Education and Applied Psychology, University of Padua, Padua, Italy

**Keywords:** Metaphor, Return to work, Motherhood, Women, Workplace, Work-life balance

## Abstract

**Purpose:**

Return to work after maternity leave represents a radical change in women’s lives. This paper aims to present a new metaphor categorization system based on two studies, which could assist working mothers in expressing the nuances of their experience when returning to work after maternity leave.

**Methods:**

We carried out the analysis of the metaphors according to the method for thematic analysis, through a multistep, iterative coding process. To ensure the researchers encode the data similarly, inter-coder reliability was achieved through the judges’ agreement method. The level of agreement between the two judges was measured by Cohen’s kappa.

**Results:**

In Study 1, we established a system comprising ten metaphor categories (namely, Natural event and/or element, Challenge and destination, Movement and/or action, Fresh start, Fight, Game and hobby, Animal, Alternate reality, Means of transport, Hostile place). In Study 2, we recognized the same metaphor categories observed in Study 1, except “Means of transport”, even with data sourced from a distinct participant group, an indicator of credibility in terms of inter-coder reliability.

**Conclusion:**

Findings highlight the usefulness of this new metaphor categorization system (named Meta4Moms@Work—Metaphors system for Moms back to Work) to facilitate a more straightforward elicitation of the meanings employed by working mothers to depict their return to work after maternity leave. Leveraging these insights, researchers/practitioners can develop and execute primary and secondary interventions aimed to enhance working mothers’ work-life balance, well-being, and mental health.

**Supplementary Information:**

The online version contains supplementary material available at 10.1007/s00737-024-01446-0.

## Introduction

As Wisława Szymborska writes “*When a child is born, the world is never ready*”. Working mothers’ (WMs) participation in the labor market is increasing. Returning to work (RTW) after maternity leave is a complex event for working women, which is often perceived as a possible source of discomfort and stress, challenging them to readjust to working life while balancing it with family life (Alstveit et al. 2011; Sabat et al. 2016; Dal Forno Martins et al. 2019). This challenge is even more difficult because, generally, they tend to have more responsibilities in both work and family (Craig 2006; Grice et al. 2011).

The present study is situated within a regulatory framework encompassing a broad spectrum of protections for WMs, as outlined in the legislative decree of 26 March 2001, n. 151. The five-month compulsory maternal leave^1^ may be followed by an additional parental leave of six months, extendable up to eleven months if the father also takes advantage of it within the first twelve years of the child’s life. These periods of absence from work are partially covered by an allowance by the National Institute for Social Security (INPS). Next, the “right of return” is established, granting workers the right to resume the same position held before the leave. Additionally, for several months after RTW, women cannot be assigned to tiring, dangerous, unhealthy, or night work.^2^ To ease a gradual RTW, further facilitations are provided, such as daily breaks or remote work. Finally, dismissal is prohibited for up to one year from the child’s birth. As for resignations, a specific procedure must be followed, requiring validation by the Inspection Service of the Ministry of Labor. Although paid and longer maternity leaves tend to be associated with a reduction of mental health difficulties (e.g., postpartum depression symptoms) in high-income countries (e.g., Italy) (Hidalgo-Padilla et al. 2023), the transition from mother to WM represents a radical change in women’s lives, because it requires integrating the mother and the worker identities (Ladge et al. 2012; Parcsi and Curtin 2013; Ladge and Greenberg 2015). These difficulties can lead women to become mothers later (Stumbitz et al. 2018) or not to RTW after maternity to devote themselves to childcare (Morgenroth and Heilman 2017; Manna et al. 2021). This decision is often influenced by the lack of public services for early childcare and the restrictive labor policies that can affect young women, such as the prevalence of horizontal and vertical job segregation and the overuse of part-time employment. In 2022, the employment rate for Italian mothers aged 25 to 54 was 56.1%, versus the childless women’s employment rate of 67% (Save the Children 2023).

Although efforts are ongoing to counteract gender stereotypes, these have proven remarkably resilient because they reflect deeply rooted beliefs about the roles that women and men should prioritize. Generally, these stereotypes characterize women as communal (i.e., affectionate, caring, kind) and men as agentic (i.e., competent, independent, dominant) (Bhatia and Bhatia 2021). Moreover, they represent oppositional stereotypes, viewing women as having a low level of agency and men as lacking communal traits (Neuenswander et al. 2023). Regarding parenthood, Valiquette-Tessier et al. (2019) observe that fathers are primarily seen as financial providers, guides, caregivers, protectors, and disciplinarians. However, the prevailing view is that of the father as the financial provider, with the caregiver stereotype never outweighing that of the breadwinner. In contrast, concerning stereotypes related to motherhood, mothers are perceived as primary caregivers, role models, and homemakers. Despite increasing women’s participation in the workforce, mothers are primarily associated with caregiving tasks. WMs who hold this stereotype have stronger feelings of guilt associated with full-time work and overtime than those who hold more egalitarian beliefs regarding women and men in the workplace (Aarntzen et al. 2023).

These gender stereotypes can lead to a gendered division of mental labor. A recent review (Reich-Stiebert et al. 2023) suggests that women are more likely to be considered responsible for performing most of the mental work involved in family management compared to men. Additionally, the perception of mental work differs between men and women: men believe they share mental work equally with their partners, while women perceive themselves as solely responsible for such activities, expressing greater concern for childcare and their health issues. This mental burden can be perceived as exhausting and draining, leading to a decrease in well-being and an increase in emotional distress. Furthermore, it can impact paid work by influencing career choices and impairing performance due to reduced concentration.

Gender stereotypes persist within organizations as well. Unfortunately, a stereotypical view of the “ideal worker” (Williams 2000) still exists, discriminating between “good” and “bad” workers. According to this view, the ideal worker is fully dedicated to their job, has no children, or if they do, has someone at home taking care of them so that they can work full-time and even overtime. This results in a negative perception of WMs, who are not considered ideal workers (Hampson 2018). While motherhood is perceived as a joyful state outside organizations, within them, a stereotypical view prevails, associating it with fewer opportunities and work-related experiences and reduced performance capacity compared to childless workers. Moreover, in organizational contexts where the ideal worker norm is pervasive, access to family-friendly strategies, especially for WMs, is often linked to a perceived lack of dedication to work. According to this stereotypical view of the ideal worker, beyond compulsory maternal leave, WMs are thought not to need additional accommodations or permissions related to their children (Greer and Morgan 2016).

Therefore, cultural norms and shared expectations preserve negative beliefs about women, their personal lives, and careers, leading to myths negatively affecting women’s decision-making (Sockol and Battle 2015; Thomason et al. 2015). Furthermore, if not managed properly, RTW can have negative consequences for both WMs (e.g., low levels of job satisfaction and earnings) and organizations (e.g., high turnover costs) (Coulson et al. 2012; Carluccio et al. 2020). Organizations are therefore called to understand how RTW is perceived by WMs and to intervene accordingly, supporting them by promoting well-being and mental health and providing useful strategies for work-life balance (McCardel et al. 2022).

### Metaphors in organizations

In the past, metaphors, involving comparing one concept to another (Lakoff and Johnson 1980a) and offering a linguistic framework for individuals to comprehend and give meaning to their experience, have served as a language tool to tap into profound thoughts. Metaphors (e.g., verbal, visual) serve as powerful interpretative tools, particularly during complex and delicate experiences, such as significant changes (e.g., RTW after maternity), that make it challenging to express thoughts clearly and consciously (Tracy et al. 2006). Sometimes, a concept, based on its complexity, may be formed by several metaphorical elements combined coherently. The various metaphorical structures highlight different facets of one concept (Lakoff and Johnson 1980b). In our perspective, metaphors arise from the interweaving of images and implications, whose message can only be grasped in context and can change when in a new configuration (Pelletier 1978).

In applied research fields, such as work and organizational psychology, metaphors can be effectively used to gain insight into workers’ perceptions and experiences. Metaphors are not only the object of research but also tools that encourage adopting different points of view (Smollan 2014; Manuti and Giancaspro 2021; Dal Corso et al. 2021; Schoeneborn et al. 2022; Grangeiro et al. 2022; Bracco and Ivaldi 2023). Metaphors assume this pivotal role due to their ability to offer linguistic flexibility and expressiveness, thereby playing a crucial part in the sense-making processes of top managers, strategists, entrepreneurs, middle managers, administrators, and blue-collar workers (Cornelissen et al. 2008). Furthermore, the utilization of metaphors is appealing because of their capacity to foster employees’ reflexivity and provide opportunities for uncovering insights that may not be readily apparent in more constrained questioning. The collective examination of identified metaphors among workers can also stimulate the collaborative development of their understanding, fostering an appreciation for individual differences in perception within their specific context (Scott and Armstrong 2019).

This paper aims to offer a new metaphor categorization system through two studies to facilitate WMs elicit meanings to describe their RTW after maternity leave.

## STUDY 1

### Methods

#### Participants

Participants were recruited using a convenience sample in healthcare and were eligible to participate if aged 18 years or older and having experienced RTW after maternity leave. If mothers had prior children, they reported on the most recent RTW experience. Participants were 132 WMs living in a Northern Italian urban area. Table 1 shows the participants’ socio-demographics.

**Table 1:** WMs’ sociodemographics

Sociodemographics	Percentage
Age	
Between 31 and 40 years old	58.7%
41 and over	32.6%
Between 18 and 30	8.7%
Occupation	
Hospital ward staff	78.1%
Administration/management personnel	12.1%
Doctor	9.8%
Time of work	
Full-time	59.1%
Part-time	34.1%
No reply	6.8%
Work contract	
Open-ended contract	97.7%
Fixed-term contract	2.3%

^a^The translation of the WMs’ words has been as faithful as possible – retaining colloquial expressions and grammar imperfections

#### Measures and procedure

Participants were administered a paper-based self-report questionnaire in the healthcare organization where they worked. At the beginning of the self-report questionnaire, participants were invited to describe metaphorically, in writing, their RTW after the maternity experience by completing the sentence “*My RTW was* … *because* …”. Socio-demographic information was collected at the end of the questionnaire. Each participant was identified by an ID number to aggregate data and preserve anonymity. Instructions to participate in the study and complete the questionnaire were delivered to each worker separately.

#### Data analysis

The metaphors were analyzed according to the method for thematic analysis by Braun and Clarke (2006, Braun et al. 2019), useful for identifying, analyzing, and reporting patterns (themes) within data. This analysis was carried out through a multistep, iterative coding process. Two independent researchers read through the data many times, integrating their field notes. Data were uploaded to Atlas.ti 23 (ATLAS.ti Scientific Software Development GmbH 2023) to assist with data organization. The researchers first coded the metaphors, as well as the participants’ subsequent explanations. Each metaphor was coded as a unique piece of data. The next coded metaphor was then compared with the previous codes and data: if it corresponded to one of the previous codes it was coded into it, if not, a new code was created. During this phase, some responses were eliminated from the dataset because they did not contain metaphors related to RTW after maternity leave. The researchers then compared coding, working out discrepancies, and further refining the codebook. Then they looked for patterns among coded metaphors, and how they logically cohered. Once the combination of the codes (i.e., metaphorical elements) in patterns or themes (i.e., metaphor categories) was carried out, agreements and disagreements were recorded, and a third researcher tried to reach an agreement in the cases of different categorizations, to determine the assignment to an exclusive category. Finally, we verified that the identified metaphor categories supported the data, and provided a clear definition of each category.

### Results

Table 2 shows ten categories of the metaphor categorization system (named Meta4Moms@Work – Metaphors system for Moms back to Work), the frequency with which they were used, and some examples of metaphors and their metaphorical elements. The definition and meaning of each category follows.

**Table 2:** Metaphor category of Meta4Moms@Work

Metaphor category and frequency	Example metaphors reported by WMs *My return to work was … because …*
RTW is a **natural event** and/or a dynamic and unpredictable **element** (36)	*Like crossing a ****stormy sea***^***a***^*, because at times difficult but exciting*^a^. (ID: 6) *A ****storm****, because ****wind****, ****thunder,**** and ****lightning**** suddenly hit my life*. (ID: 97) *A breath of ****fresh air****, because after many months at home, I felt the need to go back to my usual life*. (ID: 98) *Being in the ****rain**** without an umbrella because there is no way you can shelter yourself from the many requests that are made of you at work and at home*. (ID: 132)
RTW is a demanding and desired **challenge** and **destination** (31)	*The return ****home**** from a long ****journey**** because everyone welcomed me with joy and serenity*. (ID: 27) *A ****challenge**** that I will win because I have studied hard to be able to do my job which I love*. (ID: 28) *A ****trip****, because to organize it you must be able to fit the needs and requirements of the whole family, but, if you like your destination, you do your very best to make it happen*. (ID: 40) *A major event, a challenge to yourself, an ****exam**** to face*. (ID: 48)
RTW is a motivating, yet trying, **movement** and/or **action** (30)	*The ****Camino de Santiago****, because it is very long but a source of great joy and satisfaction*. (ID: 31) *Having a great desire ****to cook**** a special dessert but realizing that you are missing some ingredients and that you cannot go out to buy them*. (ID: 33) *A ****run**** after a long period of inactivity, because at first it’s hard and it seems like you can’t make it, but then you get back into it and there’s pleasure again*. (ID: 64) ***Running**** toward a loved one that you haven’t seen for a long time and that you want and need to hug again*. (ID: 114)
RTW is a **fresh start** of renewal requiring adaptability (18)	*An ****awakening**** after a long sleep, as I had gotten used to the slow pace of life with a newborn*. (ID: 47) *Opening the same door as always but with ****new hands****, because work on my return always seemed the same to me, but I felt completely renewed thanks to my new status as a mother*. (ID: 69) ***Taking my life back into my hands**** because motherhood had absorbed me completely*. (ID: 115) ***Restoring**** a beautiful fresco, because little by little you see the results of your work and sometimes you are even amazed*. (ID: 122)
RTW is a painful inner **fight** (12)	*A great ****wound****, for the separation from my daughter*. (ID: 18) *A**** fight for survival**** because of sleepless nights at home and at work*. (ID: 22) *Being ****thrown into a blender****, because you know how you get in, but you don’t know what you’ll be like when you get out*. (ID: 23) ***Ripping my heart out****, because my son was only four months old, and I had no choice but to go back to work for economic reasons*. (ID: 74)
RTW is a strategic and regulated **game** and **hobby** (9)	*Finding a ****piece of the puzzle**** of my life because it makes me more complete and allows me to grow as a person*. (ID: 7) *A ****puzzle**** with many tiny little pieces, I built the edge, but the worst is yet to come*. (ID: 56) *A ****game of chess****, because it never ends, and you must never lose concentration*. (ID: 99) *Getting on a ****roller coaster**** for the strong disruption and above all because even if you don’t want to, you have to wait for the end of the ride to get off*. (ID: 105)
RTW is a predator **animal** that may deceive you (5)	*The return to the ****wolves’ den****, because my colleagues had not accepted my absence for adoptive maternity, saying that I had not given notice*. (ID: 3) *Being thrown to the ****wolves**** because I have found little understanding and help*. (ID: 109)
RTW is a surreal and imaginary **alternate reality** (4)	*Resurfacing after a dive where I saw fish and ****enchanted depths****, but I felt that the gas in my cylinders was starting to decrease, and I needed to go back to the surface*. (ID: 43) *A bad ****dream**** from which, luckily, I woke up early to find that I was in my environment, and nothing had changed*. (ID: 119)
RTW is a **means of transport** that you know how to drive or you can learn (4)	*Driving my ****car**** at night on a new road, with some hills, lots of curves, but also long flat stretches* […]. (ID: 13) *Riding a ****bicycle**** because you never forget how to do it*. (ID: 89)
RTW is a dangerous and disorienting **hostile place** (3)	*Getting out of ****prison****, because I’m back to being important as a woman as well as a mother.* (ID: 68) *Wandering in the night in an ****unfamiliar city**** because I felt really lost for many months*. (ID: 118)

^a^Metaphorical element in bold

#### RTW is a *natural event* and/or a dynamic and unpredictable *element*

This category includes metaphors containing elements of nature, from the calmest and most pleasant – light, rainbow, fresh air – to the most impetuous and destructive – hurricanes, earthquakes, storms. Here, RTW after maternity leave takes on more facets.

Although WMs enjoyed the unforgettable moments associated with maternity, the strong need for RTW emerges, perceived as an opportunity to regain possession of one’s professional life and do something for oneself. RTW, allowing women to resume their working role, guaranteed a priceless sense of freedom from the exclusive maternal role.

*A rainbow after rainy days, because now my life is colorful again*. (ID: 88)

However, some WMs used terrible images of nature to describe RTW as something overwhelming and difficult to deal with. Some participants experienced a strong responsibility without feeling ready to face it. A sense of loneliness, disorientation, and helplessness also emerges, with RTW generating big changes, causing the reference points existing before maternity to be lost.

*A hurricane, because even if it is announced, one is never quite ready to face it*. (ID: 90)

WMs therefore perceive RTW as an unpredictable, uncertain, and changeable experience, like a natural element, that may surprise them, unforeseen and not always controllable because determined by external larger forces.

#### RTW is a demanding and desired *challenge* and *destination*

These metaphors highlight the RTW challenges that WMs have to face, committing themselves to their resources to reach the goal. “Challenge” and “destination” are interconnected aspects: on the one hand the challenge, the process, the path to follow with its difficulties (e.g., sports competition, exam, marathon), on the other the goal, the destination of one’s journey (e.g., travel, vacation, road).

Some WMs reported facing difficulties relating mainly to work-life balance which required great effort, and fatigue. However, thanks to a strong passion for their work, they managed to overcome them and achieve their goals.

*Joining the Olympics, because there are many disciplines, all different and each one requires training and commitment*. (ID: 120)

Furthermore, facing these challenges has allowed some participants to discover resources they didn’t think they had, allowing them to best start this new phase.

*A beautiful adventure, because it is full of discoveries and satisfaction also given by the discovery of a new strength, of a new me*. (ID: 85)

RTW is felt as an experience demanding to face with one’s strength and skills a particularly difficult situation, to reach, after a long absence, a desirable aim, such as a good life-work balance.

#### RTW is a motivating, yet trying, *movement* and/or *action*

This category groups movement and action, from the most ordinary – walking, cooking, driving – to those requiring more energy – running, jumping, swimming. Therefore, WMs actively experience RTW by acting in their environment.

Mainly, what emerges is the fatigue of facing a new phase, with ever greater difficulties, which leads WMs to be “always in a rush”, as well as the fear of not knowing what to expect and how RTW will affect their maternal role.

*Walking balanced on a tightrope, because I frequently feel the abyss under my feet*. (ID: 25)

On the other hand, the need for RTW is highlighted, as well as the satisfaction of managing it well and reconciling family and work.

*Being scared of jumping with a parachute and then discovering, once it is open, that you can enjoy a breathtaking view*. (ID: 84)

The RTW experience perceived as a movement and/or action is the fruit of a manifestation of the WM’s will. However, such movement and/or action requires a significant effort and expenditure of energy.

#### RTW is a *fresh start* of renewal requiring adaptability

This category includes metaphors highlighting a before and an after (e.g., new chapter, awakening). For many WMs, RTW is a new phase: no longer just workers, nor just mothers, but mothers and workers together. From the metaphors, it emerges that the maternal role was not sufficient, and the need to work was felt.

*Starting a new life because everything revolves around you*. (ID: 41)

Despite some fear, WMs faced it with curiosity and enthusiasm, because for many work represented the place to assert themselves and give their best.

*Exploring new territories because there was so much curiosity and work to be discovered*. (ID: 62)

The WM experiences RTW as something different, an experience of renewal compared to her pre-maternity condition; a situation in which she is newly fulfilling a new work and family role requiring profoundly transformed behaviors, flexibility, and adaptability.

#### RTW is a painful inner *fight*

As already outlined, RTW can be perceived as enriching, thanks to the integration of the two roles, but tiring, due to its numerous difficulties and the complex management of work-life balance. These metaphors describe RTW in its complexity: a battle where they have suffered, been injured, and lost something (e.g., bombing, wound, heart ripping out). The first emotion to emerge is the sense of guilt linked to the separation from one’s child.

*An internal battle, because the sense of guilt still struggles with my desire for a career today*. (ID: 91)

For the participants RTW also represented breaking the habits established during the months of maternity leave, leading to uncertainties about how their lives would continue.

*A sentence to forced labor, because there are times in the hospital when I really feel like I’m in a ball-and-chain situation*. (ID: 101)

WMs, therefore, attribute to RTW the meaning of a conflictual experience, like a fight, a strong inner conflict where the woman is split between what she is told is right and what she feels like doing for her baby and work. At the same time, it is experienced as painful, a source of worries for possible complications and negative consequences (e.g., connected to separation from her baby, missing out on a promotion) distressing or upsetting WMs deeply.

#### RTW is a strategic and regulated *game* and *hobby*

These metaphors depict playful and leisure activities, from classic board games (e.g., puzzle, chess) to outdoor activities (e.g., swing, roller coaster).

Participants described RTW as difficult to manage, especially concerning work-life balance, which required physical and cognitive resources.

*Composing a puzzle, because returning to work was a game of skill in matching family management with work activity, without being able to count on external family members*. (ID: 1)

Furthermore, once again, participants report a mix of contrasting and fluctuating emotions, feelings, and/or moods: on the one hand the need to start working again, on the other the sense of guilt for temporarily leaving the family.

*A swing, for the alternation of joy at being back in the world of adults and discomfort and fatigue for everything I left at home and had to do at home*. (ID: 80)

RTW is experienced as requiring acting with strategy and ingenuity, alone or in cooperation (e.g., social support from colleagues and supervisor, sharing the care load with the partner) and/or a competition with others (e.g., work-family backlash), knowing the rules of the game.

#### RTW is a predator *animal* that may deceive you

This category comprises metaphors referring to animals (e.g., wolf, fish). Although not all animals are dangerous or described with a negative connotation, in our study WMs reported animals’ most dangerous aspects. Specifically, some describe RTW as seemingly peaceful, but colleagues showing hostility and little understanding.

*The meeting between Little Red Riding Hood and the wolf, because, though apparently calm, the wolf is ready to eat you*. (ID: 104)

In the RTW experience WMs may feel like preys in the animal world, trapped or cheated.

#### RTW is a surreal and imaginary *alternate reality*

These metaphors describe RTW as something beyond normal reality, highlighting otherworldly, fantastical, alternative aspects (e.g., dream, enchanted backdrop).

Participants experienced the work environment they returned to as different from what they had left, almost unrecognizable and difficult to manage.

*I felt like an alien, because what had been my world up until a year before now seemed like another planet to me*. (ID: 65)

The RTW experience evokes a surreal and imaginary dimension, a hypothetical universe separate and different from the one where the WM lives, an illusory state of happiness, dream, contemplation, or like an abrupt return to reality.

#### RTW is a *means of transport* that you know how to drive or you can learn

This category includes metaphors referring to the use of means of transport (e.g., car, bicycle). Some participants reported that RTW, though representing a new experience with unknowns and difficulties, was familiar, automatic, as if they had resumed working from where they had left off.

*Bicycling, because I didn’t feel I had been home more than a year*. (ID: 73)

WMs experience RTW as an something they feel they can manage, control, and govern. However, to follow the desired direction, some abilities are needed that can be learnt contextually. Otherwise WMs may run the risk of losing control of their lives as mothers and workers.

#### RTW is a dangerous and disorienting *hostile place*

The last category involves metaphors describing RTW as a dangerous, hostile place, where WMs do not feel safe and/or at ease (e.g., prison, unknown city). Participants highlighted a sense of disorientation typical of a new phase of life, reporting difficulties in managing work and family.

*A great labyrinth, because some days, after hours and hours of work, it seems to me that the exit that separates me from my baby is still far away and hidden*. (ID: 87)

RTW is therefore perceived as a complex experience, often lived as there were no way out, with dangers and inextricable difficulties, that may disorient and unsettle the WM, and have her temporarily lose the clarity of mind needed to face the situation.

#### Metaphors and emotions

Some participants reported emotions, feelings, and/or moods within the metaphors. Some fluctuations are expressed: if, on the one hand, WMs reported the discouragement, fear, and awe of reconciling life and work, and the sense of guilt, abandonment, and loneliness in leaving their child, on the other hand, they also reported satisfaction, joy, and happiness in regaining possession of their professional life, emphasizing the love for their work.

Some sentences do not fit into any of the metaphor categories described as they either depicted only emotions, feelings, and/or moods (N = 6) or did not contain any metaphorical element (N = 4). Some participants (N = 14) provided no responses.

*A moment of joy because my profession satisfies me and makes me happy*. (ID: 45)

## STUDY 2

### Methods

#### Participants

Participants were recruited using a convenience sample of women working for public and private organizations, and/or freelancers. Eligibility for the study was established for individuals aged 18 or older who had experienced RTW after maternity leave. If mothers had prior children, they reported on the most recent RTW experience. Participants were 41 WMs living in a Northern Italian urban area. Table 3 shows the participants’ socio-demographics.

**Table 3:** WMs’ sociodemographics

Sociodemographics	Percentage
Age	
Between 31 and 40 years old	70.7%
41 and over	22%
Between 18 and 30	7.3%
Occupation	
Employed in paid work	90.2%
Freelancer	4.9%
Other (e.g., both employed in paid work and freelancer)	4.9%
Time of work	
Full-time	73.3%
Part-time	26.7%
Work contract	
Open-ended contract	90.2%
Fixed-term contract	7.4%
No reply	2.4%

#### Measures and procedure

All measures and the general procedure were identical to those adopted in Study 1, except (1) Study 2 is part of a larger project including participation in a psychological intervention aimed to improve the quality of RTW by reducing rigid maternal beliefs and learning techniques for managing anxiety and stress, and (2) participants were administered an online self-report questionnaire.

#### Data analysis

The analysis of the metaphors was carried out with the same methodology of Study 1. The level of agreement between the two researchers was measured by Cohen’s k index (1988). In this study, each metaphor can contain up to three metaphorical elements to be classified into different categories. Therefore, three k tests were conducted, one for each metaphorical element identified. According to Landis and Koch (1977): k < 0.00 = poor strength of agreement; 0.00 < k ≤ 0.20 = slight strength of agreement; 0.21 < k ≤ 0.40 = fair strength of agreement; 0.41 < k ≤ 0.60 = moderate strength of agreement; 0.61 < k ≤ 0.80 = substantial strength of agreement; 0.81 < k ≤ 1.00 = almost perfect agreement.

### Results

We identified the same metaphor categories that emerged in Study 1, except the “Means of transport” metaphor category. Table 4 shows some examples for each category.

**Table 4:** Distribution of metaphor category of Meta4Moms@Work

Metaphor category and frequency	Example metaphors reported by WMs *My return to work was … because …*
RTW is a **natural event** and/or a dynamic and unpredictable **element** (13)	*A ****hurricane***^***b***^* of emotions because I feel guilty for leaving the little one; worried about whether she will eat, sleep, and be well; anxious and paranoid.* (ID: 20) *A ****bolt**** from the blue because the amount of work and the organization of the new family struck and floored me, I wasn’t ready. Then, after a few weeks, we managed to find a new set-up and the ****sky cleared**** again.* (ID: 16)
RTW is a motivating, yet trying, **movement** and/or **action** (13)	*A ****run on a well-known route**** because I was immediately assigned a complex activity but which I know well and have flexibility in terms of both hours and presence in the workplace*. (ID: 2) *Constantly ****jumping over obstacles**** because my role as team leader is challenged and I have to demonstrate that I am capable of doing my job by carrying out tasks that are not part of my role.* (ID: 31)
RTW is a demanding and desired **challenge** and **destination** (11)	*A ****challenge**** because I had to overcome the fears of detachment from my daughter, but the result was positive.* (ID: 36) *A ****sports competition**** because I realized that it required energy and concentration and I felt I didn’t have either at the time.* (ID: 13)
RTW is a **fresh start** of renewal requiring adaptability (10)	***Taking my life back in hand**** because I’m back as a woman and not just a mom.* (ID: 4) *The ****new chapter of a book****: I already wanted to read it, sometimes it’s enthralling and other times less so.* (ID: 14)
RTW is a strategic and regulated **game** and **hobby** (4)	*A ****roller coaster**** because it was scary but, by now, I was sitting, the ****carousel**** had started, and I could only wait for the ride to end.* (ID: 3)
RTW is a dangerous and disorienting **hostile place** (3)	*An ****endless tunnel**** because, despite my efforts, I haven’t been able to change jobs.* (ID: 1)
RTW is a predator **animal** that may deceive you (2)	*A ****lark**** mirror because it was wrapped in shiny paper to get accepted, but it wasn’t a nice gift.* (ID: 9)
RTW is a surreal and imaginary **alternate reality** (2)	*In the morning when you wake up after a good ****dream**** because you open your eyes and your heart is filled with something beautiful, but the day starts, and you see yourself again and you go back to being the same as always.* (ID: 17)
RTW is a painful inner **fight** (1)	*Boomerang because it started well, it came back ****hitting me****.* (ID: 30)

^b^Metaphorical element in bold

Although all WMs responded, some sentences in the first assignment do not fit into any metaphor category because they either expressed only emotions, feelings, and/or moods (N = 4) or did not contain any metaphors (N = 1).

*Detachment because, being used to always being with my daughter, I felt I was abandoning her.* (ID: 21^3^)

The level of agreement between the two researchers was almost perfect. This is desirable in terms of credibility because it indicates that the researchers are coding the data similarly. Specifically, relative to the first assignment of metaphorical elements to the metaphor category, k was 0.907 (*p* < 0.001). The second and third assignments of metaphorical elements, 17 and 3 respectively, tend to enrich the metaphor. The level of agreement between the two judges was 0.895 (*p* < 0.001) and 1.00 (*p* < 0.014), respectively.

For the main metaphorical elements for each metaphor category of Meta4Moms@Work, see SI1.

## Discussion and conclusion

These studies stem from the desire to help WMs talk more easily about their experience of RTW after maternity leave. Thus, we defined the Meta4Moms@Work, a tool that includes a list of elements attributable to metaphor categories aimed to encourage WMs elicit meanings to describe their RTW. In Study 1 we identified the ten metaphor categories that compose the tool (Table 2). In Study 2 we identified the same metaphor categories that emerged in Study 1 – except “Means of transport” – despite using the data from a different group of participants, an indicator of credibility in terms of inter-coder reliability (Tracy 2020).

In general, WMs describe their RTW experience as complex, at times ambivalent and emotionally charged, by using a metaphorical vocabulary expressing a wide semantic scope. In particular, RTW is experienced with the dynamism and unpredictability typical of a natural event and/or element, that may have the traits of a demanding challenge to achieve the desired life-work balance, thanks to one’s will power and despite the effort (e.g., connected to baby’s, family, and work requirements). It is also experienced as a fresh start requiring adjustment to a new situation (i.e., of working mother), that can create a painful inner conflict (e.g., related to the separation from one’s baby because of a work choice). WMs also describe it as a game requiring a knowledge of strategies and rules (e.g., asking for temporary flexitime), to avoid feeling one has no way out and is at the mercy of organizational policies that are not always virtuous. It may also be experienced as an abrupt interruption of a dream, almost an illusion experienced during maternity leave. Yet again, RTW may be experienced with the perception of managing it or the awareness of being able to learn how, otherwise it may be experienced as a danger disorienting the WM (e.g., when, while enjoying a conciliation, she suffers colleagues’ negative repercussions, the so-called backlash; Perrigino et al. 2018).

Although a fair amount of literature has explored the role of metaphors in organizational contexts, in our opinion, this paper is the first aimed to create a categorization system for the experience of RTW after maternity leave. The two studies highlight the power of metaphorical language, despite the difficulty of expressing oneself metaphorically. In our studies, we used the forced metaphor approach (i.e., asking participants to represent their experience of RTW after maternity leave through a metaphor) (Tracy 2020). Although we often use metaphors daily, even without realizing it, many are unclear about what a metaphor is, or have difficulties producing a non-trivial metaphorical expression. The limitation of this approach is especially apparent in Study 1, as evidenced by the number of non-responses or sentences not containing metaphorical elements. Future studies could collect the metaphors that WMs use to describe the experience of RTW after maternity leave also using different approaches, such as the guided one, in which the researcher develops a list of metaphors to help participants describe their experience, overcoming the difficulty of producing an original metaphorical expression. The list of metaphorical elements present in the Meta4Moms@Work may be useful for this purpose.

Another limitation is that the data derives from Northern Italian open-ended-contract employees. Future research could improve the credibility of the Meta4Moms@Work through multivocality (Tracy 2020), by taking into account a variety of points of view (e.g., age, education, ethnicity, occupation) and recognizing how differences play an important role in the diverse narratives.

The usefulness of Meta4Moms@Work is also highlighted in the researcher/practitioner-client relationship because foreseeing the use of the metaphor brings a new awareness and allows WMs to consider the lived experience from different points of view. This tool may be useful in the organizational field to elicit the meanings through which WMs represent their RTW. Based on these, the researcher/practitioner can design primary and secondary psychological interventions – individual and in small groups – aimed to promote integration between the identities of mother and worker (Ladge et al. 2012; Ladge and Greenberg 2015), reduce irrational beliefs at work (Falco et al. 2017) and about maternity (Thomason et al. 2015), strengthen positive resources and, more generally, foster work-life balance, maternal well-being, mental health, and job-related outcomes (Falletta et al. 2020). In further studies, starting from the metaphorical elements constituting the defined metaphor categories, we propose the creation of a photocard toolkit, in the tradition of the best-known methods used in clinical, social, and applied psychology (Wang and Burris 1997; Vacheret 2002; Weiser 2016; Gillian 2023), composed of photographs (i.e., visual metaphors) to be used as a stimulus to help WMs elicit personal contents and meanings relative to RTW, within an intervention based on the analysis of the client’s need and in line with the professional’s education and expertise.

### Supplementary Information

Below is the link to the electronic supplementary material.

Supplementary file1 (PDF 138 KB)

## Footnotes

1 The five months of compulsory maternal leave can be taken two months before and three months after childbirth, or, if medical conditions allow, all after childbirth.

2 Night work is prohibited not only during pregnancy, but also until the child is one year old. Subsequently, up to the child’s third year, the mother cannot be asked to work at night unless she consents.

3 In Study 2 the coding started again from ID: 1.
