# Supplementary material for: Unleashing the potential of metaphors: a categorization system for exploring return to work after maternity
Source: Arch Womens Ment Health. 2024 Feb 20;27(4):607–18. doi: 10.1007/s00737-024-01446-0 (PMC11231012; doi:10.1007/s00737-024-01446-0)
Supplement: Supplementary file 1 — (PDF 138 KB) [file 737_2024_1446_MOESM1_ESM.pdf]

TITLE:

Unleashing the Potential of Metaphors: A Categorization System for Exploring Return to Work after Maternity

JOURNAL: Archives of Women's Mental Health

AUTHORS:

Sebastiano Rapisarda[1], Valentina Santoro[1], Laura Dal Corso[1]

AUTHORS' AFFILIATION:

[1] Department of Philosophy, Sociology, Education and Applied Psychology, University of Padua, Padua, Italy

CORRESPONDING AUTHOR:

Sebastiano Rapisarda, sebastiano.rapisarda@unipd.it

Meta4Moms@Work – Metaphor system for Moms back to Work

Metaphor category	Metaphorical elements	Metaphor category	Metaphorical elements	Metaphor category	Metaphorical elements	Metaphor category	Metaphorical elements
**Natural event and/or element**	Air	**Challenge and destination**	Adventure	**Fresh start**	Awakening	**Animal**	Lark
	Atmosphere		Challenge		New chapter		Fish
	Cold		Employee on probation		Regeneration		Wolf
	Dark		Exam		Restoration		…
	Desert		Graduation		…	**Alternate reality**	Alien
	Earthquake		Home	**Fight**	Ball tossed		Another planet
	Flash		Marathon		Battle		Dream
	Fog		Movie		Blender		Enchanted backdrop
	Forest		Olympics		Bombing		…
	Heat		Road		Cutting the umbilical cord	**Means of transport**	Bicycle
	Hurricane		Sports competition		Fight		Car
	Island		Travel		Heart ripping out		…
	Landscape		Vacation		Sentence	**Hostile place**	Labyrinth
	Light		…		Space shot		Prison
	Morning	**Movement and/or action**	Climb		Wound		Tunnel
	Mountain		Cook		…		Unknown city
	Oxygen		Dive	**Game and hobby**	Boomerang		…
	Quicksand		Drive		Chess	**Other categories**	…
	Rain		Jump		Puzzle		
	Rainbow		Open		Roller coaster		
	Sea		Return		Seesaw		
	Spring		Run		Skill game		
	Storm		Swim		…		
	Thunder		Walk				
	Thunderstorm		…				
	Water						
	…[a]						

[a] Other possible metaphorical elements.
